# An integrated experimental-computational approach for predicting virulence in New Zealand white rabbits and humans following inhalation exposure to *Bacillus anthracis* spores

**DOI:** 10.1371/journal.pone.0219160

**Published:** 2019-07-01

**Authors:** Becky M. Hess, Dennis G. Thomas, Thomas J. Weber, Janine R. Hutchison, Timothy M. Straub, Cynthia J. Bruckner-Lea, Joshua D. Powell, Senthil Kabilan, Richard A. Corley

**Affiliations:** 1 Chemical and Biological Signature Sciences, Pacific Northwest National Laboratory, Richland, WA, United States of America; 2 Biological Sciences Division, Pacific Northwest National Laboratory, Richland, WA, United States of America; Laurentian, CANADA

## Abstract

Inhalation of *Bacillus anthracis* spores can lead to an anthrax infection that can be fatal. Previously published mathematical models have extrapolated kinetic rates associated with bacterial growth in New Zealand White (NZW) rabbits to humans, but to date, actual measurements of the underlying processes associated with anthrax virulence between species have not been conducted. To address this knowledge gap, we have quantified species-specific rate constants associated with germination, proliferation, and immune cell inactivation of *B*. *anthracis* Sterne using an *in vitro* test platform that includes primary lung epithelial and immune cells. The generated data was then used to develop a physiologically based biokinetic model (PBBK) which quantitatively compares bacterial growth and mean time to death under lethal conditions in rabbits and humans. Simulations based upon our *in vitro* data and previously published *in vivo* data from rabbits indicate that disease progression is likely to be faster in humans than in NZW rabbits under comparable total deposited dose conditions. With the computational framework established, PBBK parameters can now be refined using experimental data for lethal *B*. *anthracis* strains (e.g. Ames) under identical conditions in future studies. The PBBK model can also be linked to existing aerosol dosimetry models that account for species-specific differences in aerosol deposition patterns to further improve the human health risk assessment of inhalation anthrax.

## Introduction

*Bacillus anthracis* is a bacterium that causes anthrax disease [1]. It has been used in bioterrorism attacks against the United States on multiple occasions, including the 2001 Amerithrax attacks, and as part of livestock sabotage during World War I [2]. Human infections can occur from inhalation, from bacteria entering wounds in the skin, or from ingestion of the bacteria in spore form. All three types of infection can be fatal, with inhalation leading to the highest death rates [3]. Once inhaled, the spores germinate to the vegetative bacterial state and produce toxins. The vegetative bacteria can proliferate, resulting in progressive infection, increased toxin production, and death [3].

Current models predicting anthrax virulence in humans are based upon *in vivo* studies primarily using New Zealand white (NZW) rabbits as the model system [4]. However, the accuracy of using data from rabbit studies to predict human outcomes remains uncertain [5]. For example, the dose-response curve for lethality as a function of inhaled spore number in the rabbit is transformed into human-equivalent doses using existing computational 3D lung dosimetry models and species-to-species dose scaling relationships [6, 7]. Such inferences assume that humans respond the same way as rabbits at the human-equivalent doses. Although species differences for the deposited dose fraction of inhaled spores can be estimated and taken into account, accurate and reliable rabbit-to-human dose-response extrapolations require knowledge about the virulence characteristics of the pathogen in both rabbits and humans.

To characterize *Bacillus anthracis* virulence, a number of critical factors, such as the rates of spore germination, bacterial proliferation, and immune cell-dependent inactivation of bacteria must be measured. Germination and proliferation rates can determine the progression of the disease, however, the rabbit and human immune systems are capable of fighting the infection by attacking either the spores or the active bacteria (inactivation) which can significantly alter disease progression. Thus, the balance between bacterial growth (germination and proliferation) and the immune response (inactivation) are critical factors for determining anthrax disease progression. Since human *in vivo* experiments are not feasible, there is currently no knowledge about the differences in the abovementioned virulence characteristics of the inhaled pathogen between humans and rabbits.

To address this knowledge gap, we have developed a physiologically relevant *in vitro* lung tissue platform that enables direct species comparison of bacterial growth kinetics, and conducted *in vitro* infectivity studies using *Bacillus anthracis* Sterne spores. The Sterne strain is relatively avirulent, can be handled under standard BSL2 culture conditions and is used as a surrogate for the virulent Ames strain to develop the approach and computational model parameters. Growth rate and lag time parameters are similar between Sterne and Ames strains in nutrient rich environments, while the Ames strain can reach slightly higher maximum population densities [8]. Thus, the Sterne strain is a reasonable surrogate when investigating model parameters associated with spore germination and proliferation. The Sterne strain can also be used to compare the capacity of immune cells isolated from different species to inactivate spores under identical experimental conditions. However, it is noted that the Sterne strain lacks a protective capsule and may lead to an overestimation of immune cell mediated clearance of spores. To address this difference, studies using a virulent strain (e.g. Ames) are required, but are beyond our present scope. Our primary goal is to establish a computational framework that incorporates spore germination rates, lag times, proliferation rates and ultimately, immune cell clearance parameters. As such, our model system enables detailed investigation of human and rabbit cellular responses to anthrax spores in a physiologically relevant context *in vitro* using air-liquid interface (ALI) cultures which recapitulate airway epithelial biology. Quantitative data obtained from the ALI culture system is incorporated into an *in vitro* physiologically based biokinetic (PBBK) computational model to test the reproducibility of the system in characterizing the kinetics of spore germination and vegetative cell proliferation in the presence and absence of immune cell-dependent inactivation. We also combine the *in vitro* PBBK model with an existing two-compartment *in vivo* PBBK model [4] to quantitatively compare the growth of bacteria in the lung airway between humans and rabbits. These models were used to predict mean time to death (MTD) under lethal conditions in both rabbits and humans, with limitations in the model understood.

## Materials and methods

### Ethics statement

The housing and humane euthanasia of New Zealand white rabbits (2.5 to 4 kg; Charles River through Oakwood Research facility, Oxford, MI) was approved by Pacific Northwest National Laboratory Institutional Animal Care and Use Committee (IACUC: 2014–04) under Animal Welfare Assurance Number A3353-01, and by the institutional biosafety committee. We comply with USDA regulations governing animal care and usage, as well as all other relevant local, State, and Federal regulations concerning animal care and usage. The USDA registration number is 91-R-006. In addition, we have a current National Institutes of Health (NIH) assurance number for animal care and usage, this OLAW PHS Assurance number is A3353-01 and we are accredited through Association for Assessment and Accreditation of Laboratory Animal Care (AAALAC) International, unit number 000425.

### Cell lines and reagents

Isolation of primary rabbit bronchial epithelial cells, peripheral blood mononuclear cells (PBMCs) and differentiation of PBMCs into macrophages and dendritic cells was as described previously [9]. Primary normal human bronchial epithelial cells (NHBEs) cells (CC-2540, Lonza) were cultured using Bronchial Life B/T medium with LifeFactors kit (LL-0023, LifeLine Cell Technology) according to the manufacturer’s instructions. The media was supplemented with 1x primocin (NC9141851, Fisher Scientific) and Fungizone (15290–018, Gibco).

### Culturing at air-liquid interface (ALI)

Primary human or rabbit bronchial epithelial cells were seeded onto 0.4 micron permeable 12-well transwell supports (coated with 30 μg/cm^2^ rat-tail collagen; A1048301, Gibco). When cells reached 100% confluence, the cells were brought to ALI and supplemented using Air-Liquid Interface Epithelial Differentiation Medium (LM-0050, LifeLine Cell Technology) according to the manufacturer’s instructions. Medium was changed every 48 hours, leaving the apical side of cells exposed to air. Cells were cultured at ALI for 10 to 14 days prior to infection assays with *B*. *anthracis*. A visible mucus layer was observed on the apical side of the cells by day 4 of ALI culture.

### Preparation of macrophages from PBMCs

Rabbit PBMCs isolated in house and human PBMCs (PCS-800-011, ATCC) were seeded in T-75 flasks. PBMCs were differentiated into macrophages by culturing in RPMI-1640 (22400–089, Gibco) supplemented with 2 mM L-glutamine (Gibco, 25303–164), 1x sodium pyruvate (113600–070, Gibco), 1x non-essential amino acids (11140–050, Gibco) and 25 ng/mL GM-CSF (300–03, PeproTech). This media was replaced every 48 hours for six days prior to integration with the epithelial cells for the spore challenge assay.

### Preparation of dendritic cells from PBMCs

Rabbit PBMCs isolated in house and human PBMCs (PCS-800-011, ATCC) were seeded in T-75 flasks. PBMCs were differentiated into dendritic cells by culturing in RPMI-1640 (22400–089, Gibco) supplemented with 2 mM L-glutamine (Gibco, 25303–164), 1x sodium pyruvate (113600–070, Gibco), 1x non-essential amino acids (11140–050, Gibco) and 25 ng/mL GM-CSF (300–03, PeproTech), and 20 ng/mL IL-4 (200–04, Peprotech). The media is replaced every 48 hours for four days prior to integration with the epithelial cells for the spore challenge assay. On day five of culturing, the media is supplemented with 1 μg/mL PGE_2_ (P6532, Peprotech) and incubated for an additional 24 hours prior to integrating the dendritic cells with the epithelial cells for the spore infection assay.

### Preparation of cells for the spore infection assay

Approximately 18 hours prior to the spore challenge assay, the basal medium on the ALI cultures was replaced with a 50:50 blend of Air-Liquid Interface Epithelial Differentiation Medium and assay media without antibiotics present. The assay media formulation is 1x DMEM (11960–044, Gibco) supplemented with 2 mM L-Glutamine (25030–164, Gibco) and 1% species specific serum (rabbit serum, S15110H, Atlanta Biologicals; human serum, H3667-100ML, Sigma Aldrich). One hour prior to the spore challenge assay, cells were washed twice in complete assay media (15 minute incubation period for each wash) to condition the cells to assay conditions and remove antibiotics that would interfere with spore germination/proliferation.

### Spore preparation and enumeration

The avirulent *Bacillus anthracis* Sterne 34F2 (pX01^+^, pX02^-^) strain was kindly provided by Dr. David Wunschel (Pacific Northwest National Laboratory). Spore preparation and enumeration were conducted as previously described [10].

### Spore infection assays

Spores were added to the co-cultures (epithelial cells with dendritic cells and macrophages) on the apical side of the cells. At the indicated time points, the assay media and cells were harvested and transferred to 2 mL microcentrifuge tubes. The number of spores and vegetative bacteria were quantified as previously described [10].

### *In vitro* PBBK model parameter estimation

#### Data used for parameter estimation

We conducted spore challenge assays with the human and NZW rabbit co-culture systems on three separate test days with three biological replicates at each time point (n = 9). For each experiment, we also had the three sets of controls to determine maximal germination and proliferation rates, as well as inactivation capacity of the immune cells. These controls are described in Table 1. For each test day and time point, the outliers were removed using the Tukey algorithm after normalizing the plate counts by the actual spore dose. The normalized values from all three test days were then combined and any outliers in the combined set were removed using the Tukey algorithm. The final dataset of normalized values were averaged and the standard deviations were computed, and then fitted to the model using MATLAB *fmincon* function.

**Table 1:** A description of each control sample set that are run in parallel with the co-culture system during spore challenge assays.

Name of Control Set	Description	Purpose
Assay Media	Media used in all the experimental samples containing cells	Determine the effects of the media on spore germination and proliferation
Epithelial Cells Alone at ALI	Primary lung epithelial cells alone at air-liquid interface with assay media present on the basal side of the cells	Determine the effects of the epithelial cells on spore germination and proliferation
Immune Cells Alone (Submerged immune cells)	Equivalent number of macrophages and dendritic cells as in the co-culture system, maintained in assay media	Determine the inactivation capacity of the immune cells in the absence of the epithelial cells.

#### Bootstrapping for determining the 95% confidence region of model output

We created 1000 simulated datasets based on data points that were randomly selected within 20% CV of the measured values in the final dataset. After estimating the parameter values for each dataset, we removed those that contained values below the 2.5^th^ and above the 97.5^th^ quantile values of each parameter. The model output based on the selected parameter sets were then computed to determine the 95% confidence region.

## Results

### Development of the *in vitro* PBBK model for NZW rabbits and humans

The *in vitro* PBBK model consists of a kinetic growth rate equation for the spore (*S, Eq 1*) and for the vegetative bacteria (*V, Eq 2*):

$$\frac{dS}{dt}=-k_{g}(S-S_{s})$$ (1)

and,

$$\frac{dV}{dt}=k_{g}(S-S_{s})+\frac{B}{1+K_{I}}\left(\frac{C}{C+exp(-\frac{B}{1+K_{I}}t)}\right)V-\mu_{V}V$$ (2)

The model variables (S, V) and parameters (*k*_*g*_,*S*_*s*_,*B*,*C*,*K*_*I*_,*μ*_*V*_) are described in Table 2.

**Table 2:** Parameters for the *in vitro* PBBK model that are measured directly using the *in vitro* experimental lung platform.

Type	Symbol	Description	Units	Units Symbol
Variable	***S***	Total number of spores as a function of time	Spore count	Spore
Variable	***V***	Total number of vegetative bacteria as a function of time	Vegetative bacteria count	CFU
Parameter	***S***_***s***_	Total number of spores that fail to germinate	Spore count	Spore
Parameter	***k***_***g***_	Spore germination rate	Per hour	h^-1^
Parameter	***B***	Maximum specific growth rate of vegetative bacteria	Per hour	h^-1^
Parameter	***C***	This parameter characterizes the influence of the environment on the physiological state of the pathogen for growth.	Ratio of a per cell concentration of a critical substance needed for growth to the Michaelis-Menten constant associated with the production of the substance from enzymatic reactions; dimensionless magnitude (0 < C)*	N/A
Parameter	***K***_***I***_	Pathogen growth inactivation by the immune cells in the co-culture system.	Capacity (immune cell capacity to inactivate *B*. *anthracis* per unit time); dimensionless magnitude	N/A
Parameter	***μ***_***V***_	Death rate of vegetative bacteria.	Per hour	h^-1^

*For additional information about the parameter development, refer to Baranyi [11].

### *In vivo* PBBK model for NZW rabbits and humans

**Fig 1:**
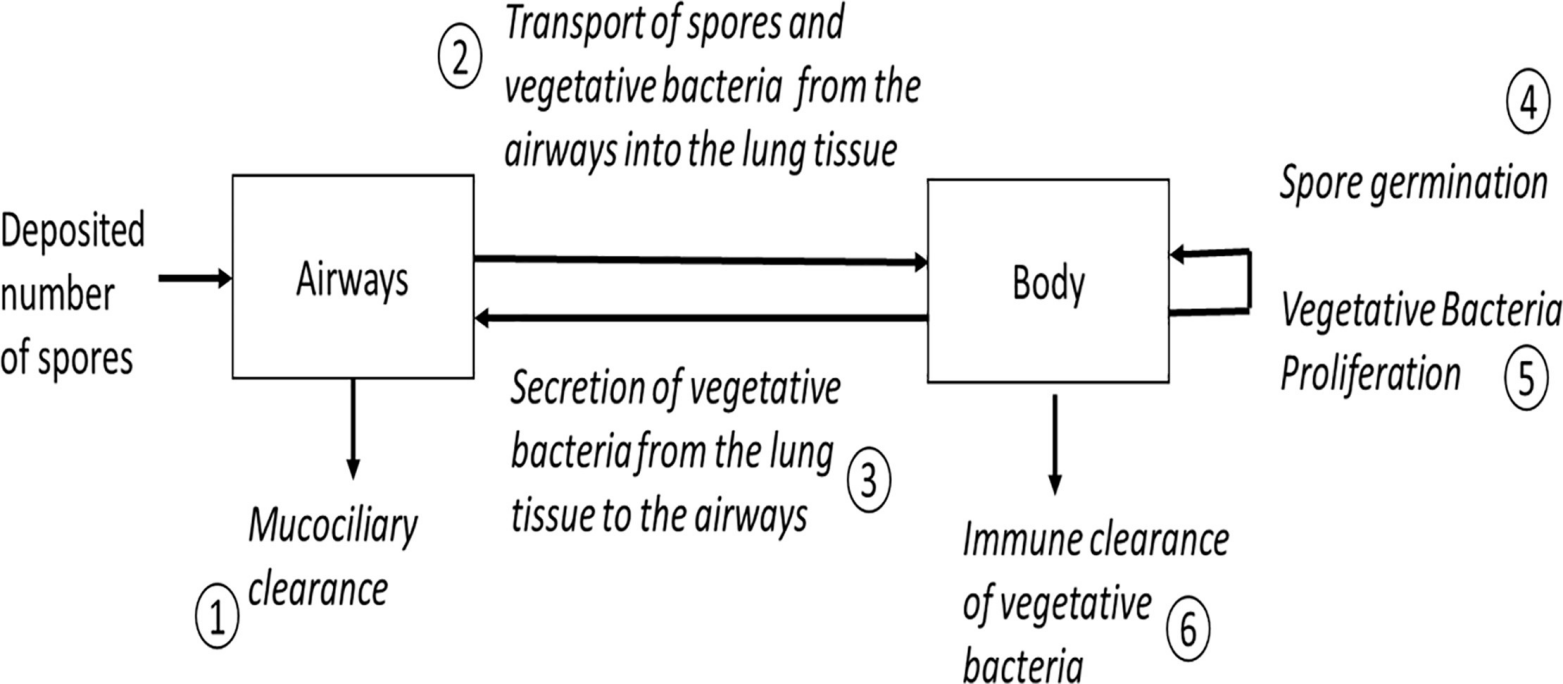
Processes described in the two-compartment *in vivo* PBBK model.

The equations of the *in vivo* PBBK model were developed based upon a published model for NZW rabbits, which is a two-compartment model for predicting the change in total bacteria in the airways and in the remainder of the rabbit body [4]. The original PBBK model does not distinguish between spores and vegetative bacteria, and hence, does not capture the species-specific kinetics for spore germination, vegetative cell proliferation, and the inactivation/clearance processes. Therefore, we replaced the bacterial growth kinetics term in the original two-compartment *in vivo* PBBK model with experimentally based biokinetic terms describing the fate of both spores and vegetative bacteria. Therefore, the refined *in vivo* PBBK model captures six biological processes to describe the time-dependent changes in the number of spores and vegetative bacteria in the airway and body (defined by Gutting et al. [4] as lung tissue, lymph nodes and blood) compartments following inhalation exposures of rabbits and humans to *Bacillus anthracis* spores. The six processes, as depicted in Fig 1, are: 1) mucociliary clearance, 2) spore and vegetative bacteria transport to lung, 3) lung tissue secretion of vegetative bacteria into the airways, 4) spore germination in the body, 5) proliferation of vegetative bacteria in the body, and 6) immune clearance of vegetative bacteria in the body. While both toxemia and septicemia are relevant mechanisms that contribute to disease progression and death, in this study we are combining both processes. These processes could be determined independently in future work.

The final equations of our *in vivo* PBBK model for the spores and vegetative bacteria in the airway and body compartments, as follows:

$$\frac{dS_{a}}{dt}=-k_{mc}S_{a}-k_{trams}S_{a}$$ (3)

$$\frac{dS_{b}}{dt}=-k_{g}S_{b}+k_{trans}S_{a}$$ (4)

$$\frac{dV_{a}}{dt}=-k_{mc}V_{a}-k_{trams}V_{a}+k_{sec}V_{b}$$ (5)

$$\frac{dV_{b}}{dt}=k_{g}S_{b}+\frac{B}{1+K_{I}}\left(\frac{C}{C+exp(-\frac{B}{1+K_{I}}t)}\right)V_{b}+k_{trans}V_{a}-k_{k}V_{b}-k_{sec}V_{b}$$ (6)

The model variables (*S*_*a*_, *V*_*a*_, *S*_*b*_, *V*_*b*_) and parameters (*k*_*mc*_,*k*_*trans*_,*k*_*sec*_,*k*_*g*_,*B*,*C*,*K*_*I*_,*k*_*k*_) are described in Table 3. *Eq* 3 describes the rate at which the number of deposited spores in the lung airway (*S*_*a*_) decreases over time due to mucociliary clearance (first term) and transport into the lung tissue (second term). *Eq* 4 describes the rate at which the number of spores in the body (*S*_*b*_) *decreases* due to germination (first term) and increases due to transport from the airway compartment (second term). Bacteria secretion (third term in *Eq* 5) leads to the presence of vegetative bacteria in the airway compartment (*V*_*a*_), which are also subject to mucociliary clearance and airway-lung transport processes (first and second terms in *Eq* 5). The first three terms in *Eq* 6 describe the rates at which the number of vegetative bacteria in the body (*V*_*b*_) increase due to germination, proliferation and airway-lung transport processes, respectively. The last two terms describe the rates at which it decreases due to bacteria clearance by immune cells and secretion into the airway from the blood.

**Table 3:** Variables and parameters for the *in vivo* PBBK model. The parameters derived from the *in vitro* experimental system are *K*_*g*,_ *B*, *C*, and *K*_*I*_.

Symbol	Description	Units	Unit Symbol
***S***_***a***_	Number of deposited spores in the lung airway	dimensionless	N/A
***S***_***b***_	Number of spores in the body	dimensionless	N/A
***V***_***a***_	Number of vegetative bacteria in the lung airway	dimensionless	N/A
***V***_***b***_	Number of vegetative bacteria in the body	dimensionless	N/A
***k***_***mc***_	Mucociliary clearance rate	Per hour	h^-1^
***k***_***trans***_	Spore transport rate into the body	Per hour	h^-1^
***k***_***sec***_	Secretion rate of vegetative bacteria from lung into the airways	Per hour	h^-1^
***k***_***g***_	Spore germination rate	Per hour	h^-1^
*B*	Maximum specific growth rate of vegetative bacteria	Per hour	h^-1^
*C*	This parameter characterizes the influence of the environment on the physiological state of the pathogen for growth.	Ratio of a per cell concentration of a critical substance needed for growth to the Michaelis-Menten constant associated with the production of the substance from enzymatic reactions; dimensionless magnitude (0 < C)*	N/A
***k***_***I***_	Pathogen growth inactivation by the immune cells in the co-culture system	Capacity (immune cell capacity to inactivate *B*. *anthracis* per unit time); dimensionless magnitude	N/A
***k***_***k***_	Immune cell clearance rate of vegetative bacteria	Per hour	h^-1^

*For additional information about the parameter development refer to Baranyi [11].

### Reproducibility in the human experimental system

**Fig 2:**
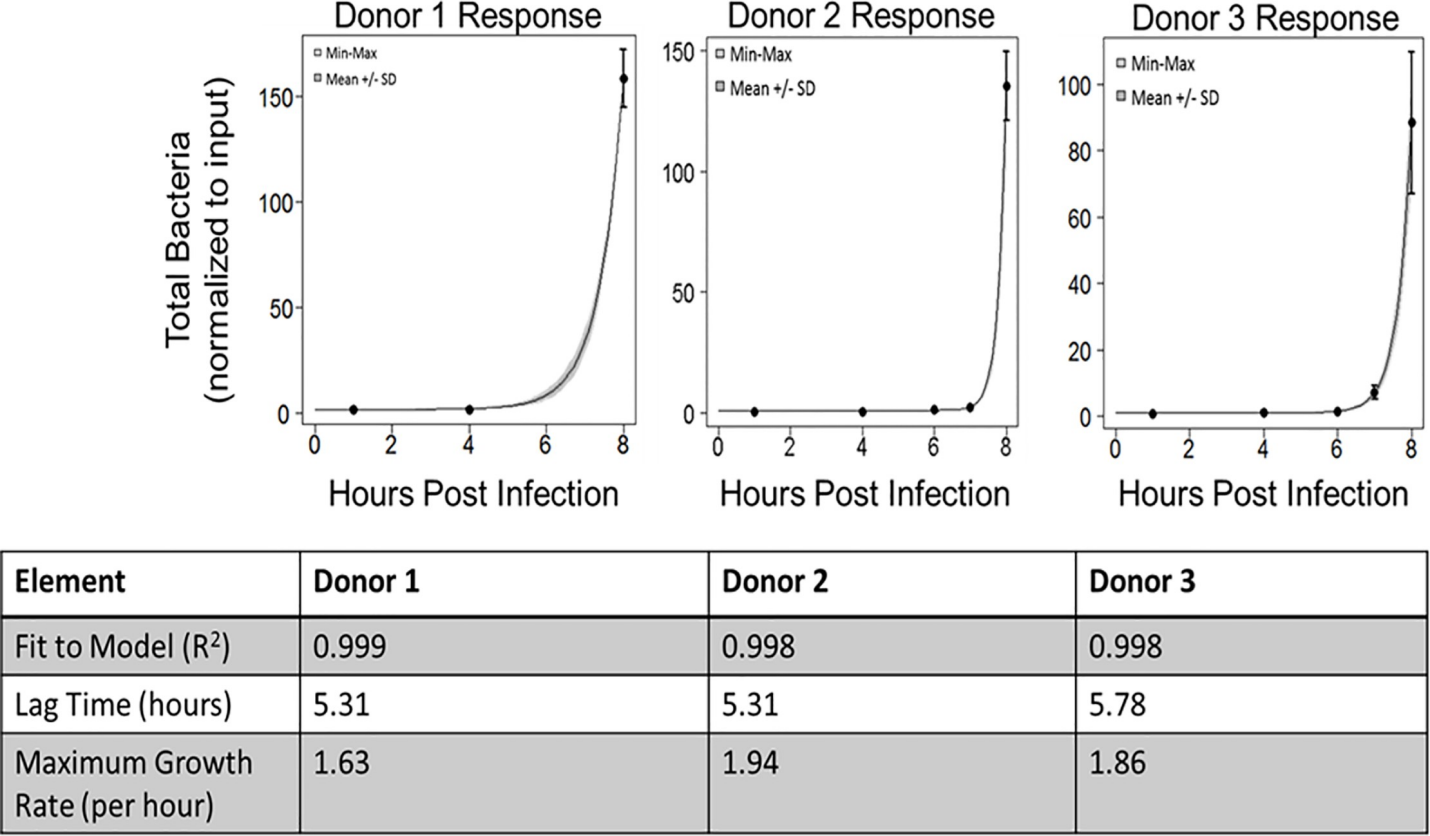
Human response from three different donors (epithelial alone at ALI challenged with *B*. *anthracis* Sterne) are reproducible and have an excellent fit (R^2^ >0.99) to the predictive growth model. Each donor was tested with three biological replicates at each of the indicated time points (n = 3). Results of a t-test showed no significant difference in lag time or maximum growth rate between donors (p > 0.05).

Experimental reproducibility was determined based on the best-fit parameter values of the *in vitro* PBBK model and the goodness of fit. For example, Fig 2 shows data collected for human bronchial epithelial cells from three different donors. The model fit shows there is excellent reproducibility between three different donors with respect to lag time and maximum bacterial growth rate. The R^2^ values for each donor were at least 0.99, and there was no significant difference in lag times or bacterial growth rates between donors. Taken together, these data indicate a highly reproducible response of human cells from different donors in the spore challenge assay.

### Testing ratios of epithelial cells to immune cells

**Fig 3:**
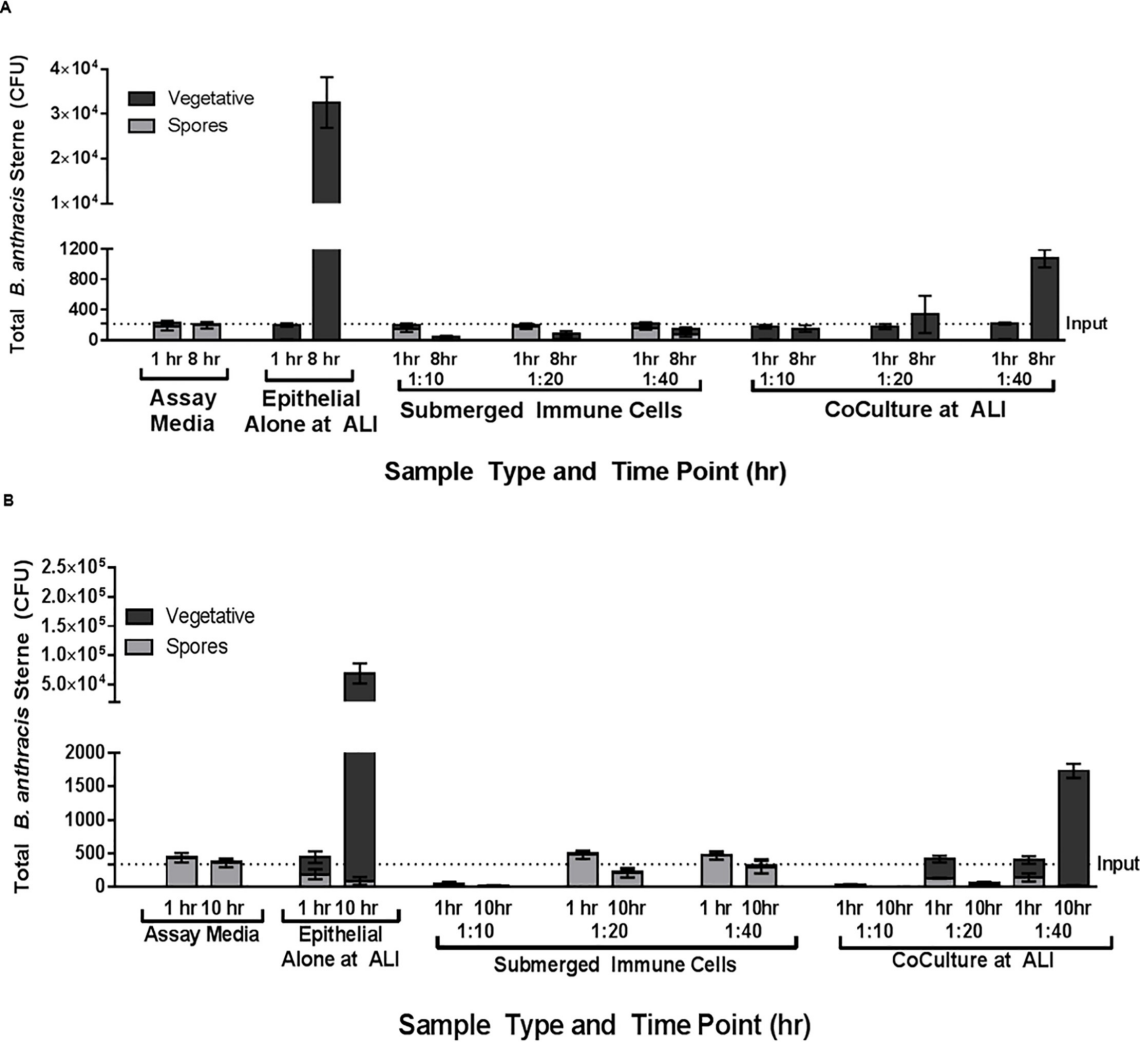
**Spore challenge assays in the human (A) and NZW rabbit (B) experimental platforms with decreasing ratios of immune cells:lung epithelial cells.** The data demonstrate that for both species, spore germination/proliferation is dependent on the presence of epithelial cells and the endpoint measured (CFUs) is decreased in immune cell/epithelial co-culture experiments as the fraction of immune cells is increased.

**Fig 4:**
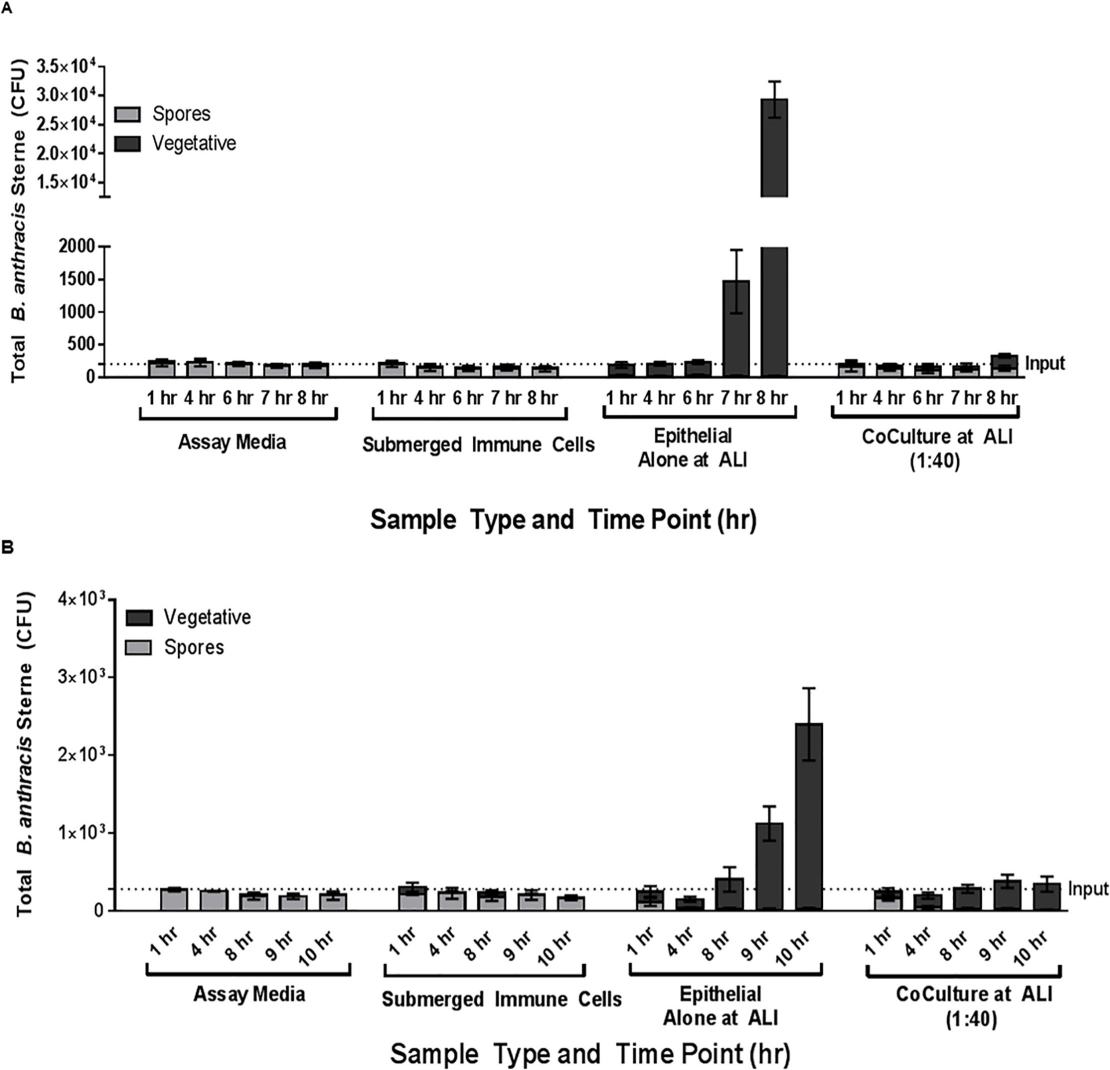
**Representative kinetic data sets from the co-culture spore challenge assay experiments with human (A) and NZW rabbit (B) experimental lung platform**s. This example shows spore challenge data for a co-culture ratio of 1:40 (immune cells:epithelial cells) at the ALI, as well as with the control experiments that were conducted along with each spore challenge assay data set (assay media alone, submerged immune cells, and epithelial cells alone at ALI).

Currently, there is a paucity of published reports that have investigated host response to anthrax using lung co-culture systems maintained at ALI. The immune cell:epithelial cell ratio is an important consideration in the development of *in vitro* model systems focused on predicting human or experimental animal responses. A normal healthy lung is estimated to contain 1 macrophage to 40 epithelial cells (discussed in [12]), and conditions of lung inflammation will have considerably higher numbers of macrophages. In order to span conditions of inflammation to normal physiology, we conducted spore challenge assays at macrophage:epithelial ratios of 1:10, 1:20, and 1:40. We observed significant germination and proliferation of *B*. *anthracis* in the presence of bronchial epithelial cells at the air liquid interface, with decreasing amounts of vegetative cells present when immune cells are included in a co-culture at the ALI (Fig 3). The kinetics of spore germination/proliferation are slightly different in rabbit and human platforms (see Fig 4) and two time points were chosen for each system (1 and 8 hr for human; 1 and 10 hr for rabbit) to enable distinction between spore and vegetative forms. We observed that at 8 and 10 hours for human and rabbit, respectively, these time points reflect when log phase growth is in effect (i.e., in the steep point of the log phase curve). These time points were chosen because at later time points (up to 24 hours post infection), the system became saturated with bacteria, with no discernible increase in the number of bacteria present (data not shown). Spores placed in culture medium alone or co-cultured with immune cells alone showed little or no increase in colony forming units (CFU), relative to the input number of spores indicated by the dotted line. The presence of immune cells clearly reduces CFU counts, relative to epithelial cells alone exposed to the same spore number.

### Kinetics of spore germination and proliferation in co-culture systems

**Fig 5:**
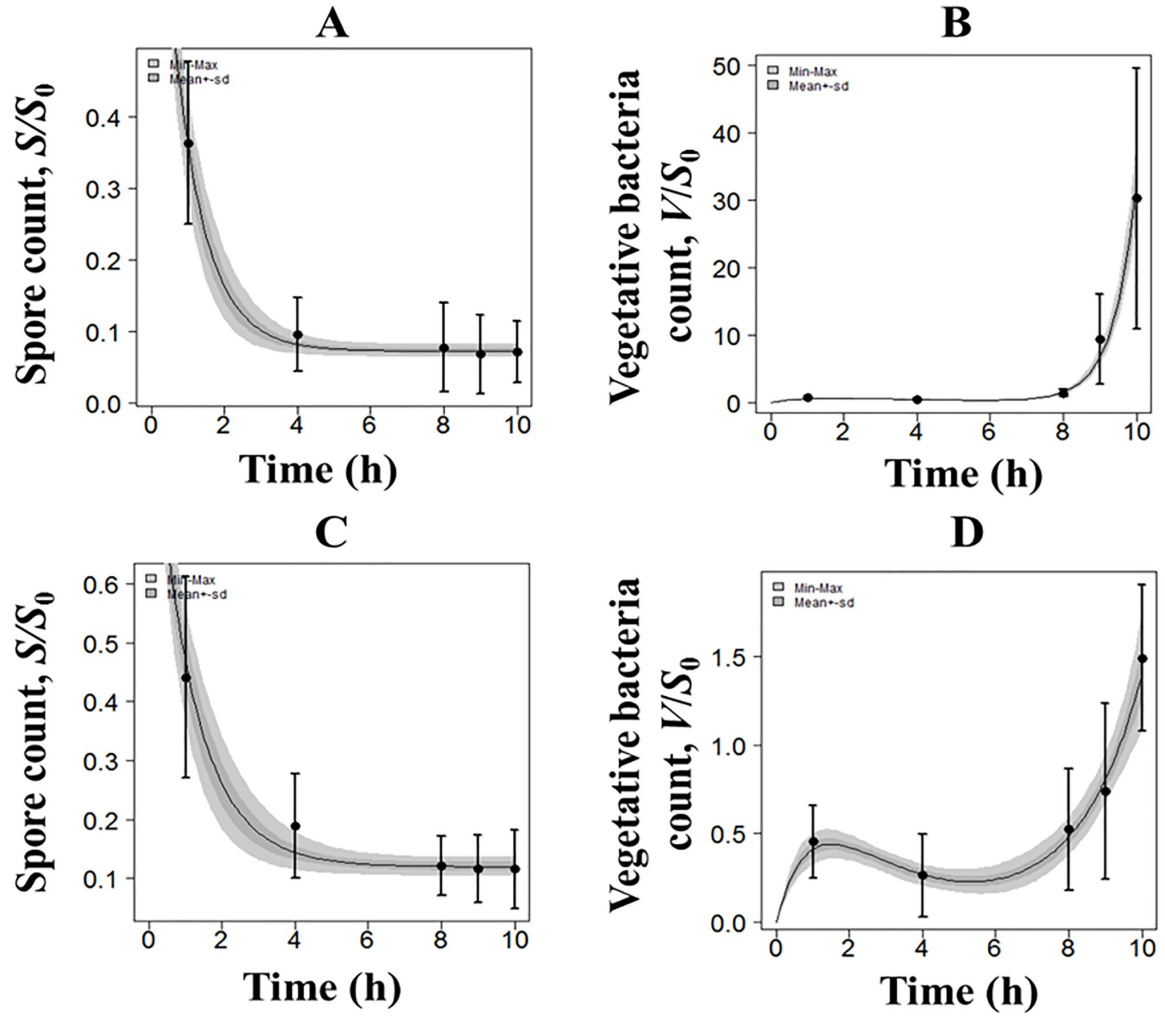
*In vitro* PBBK model fits and data for the NZW rabbit system. Spore and vegetative bacteria spore count (normalized by actual spore dose, *S*_0_) versus time in epithelial cells alone (A and B) and in co-culture (C and D); R^2^ = 0.982 and 0.740, respectively. Lines represent model fits and symbols (with error bars) represent experimental data. The shaded region shows the bootstrap 95% confidence region of the model output.

**Fig 6:**
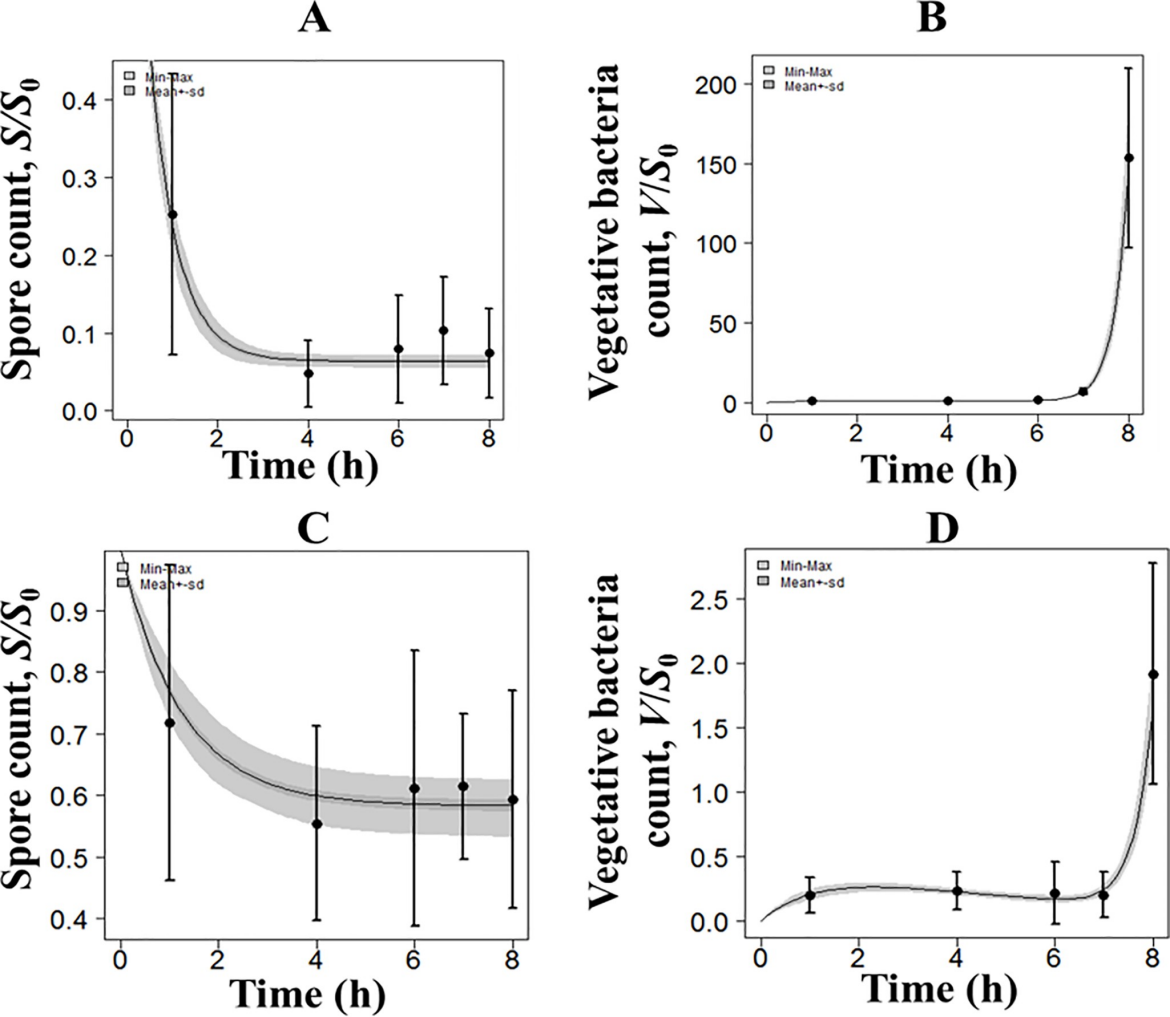
*In vitro* PBBK model fits and data for the human system. Spore and vegetative bacteria spore count (normalized by actual spore dose, *S*_0_) versus time in epithelial cells alone (A and B) and in co-culture (C and D); R^2^ = 0.997 and 0.965, respectively. Lines represent model fits and symbols (with error bars) represent experimental data. The shaded region shows the bootstrap 95% confidence region of the model output.

Representative datasets describing the kinetics of spore germination/proliferation occurring in the co-culture systems is shown in Fig 4. Results from kinetic experiments at the immune cell:epithelial cell ratio expected for healthy lungs (1:40) were used to develop the *in vitro* PBBK model shown in Figs 5 and 6.

The parameter values that were the best-fit to the *in vitro* PBBK model output and the data for the NZW rabbit and human ALI systems at 1:40 are listed in Table 4. The corresponding best-fits are shown in Figs 5 and 6, respectively. The parameter values of the spore germination and proliferation processes (*k*_*g*_,*B*,*C*) were first estimated by fitting the data from the epithelial cells alone at the ALI. These values were fixed when fitting the co-culture data set to determine the inactivation capacity (*K*_*I*_) of the co-culture system for *B*. *anthracis*. The good agreement between the model fits and the data (Figs 5 and 6) confirms that the equations of the model correctly describe the bacterial growth kinetics in our *in vitro* ALI lung tissue system. Although the R^2^ value of model fits for the rabbit co-culture system is lower than that for the human system (0.7 versus 0.97), the *in vitro* model clearly captured the time-dependent behaviors of spores and vegetative bacteria in epithelial-only and immune cell:epithelial cell co-culture ALI systems for both species. In all cases, the predicted values are well within the experimental error bars, and the model is able to correctly capture the rate at which the number of spores and vegetative bacteria change over the time period of observation. The results show that the germination rate, *k*_*g*_, is 0.74 times lower and the maximum proliferation rate, *B*, is 1.72 times higher for the human system compared to the rabbit system.

**Table 4:** *In vitro* PBBK model parameter estimates for NZW rabbit and human co-culture systems. Values in parentheses are the 95% confidence intervals obtained from 1000 bootstrap runs.

Parameter	Rabbit		Human	
	EPI-alone*	Co-culture**	EPI-alone	Co-culture
***S***_***s***_ **(*S***_**0**_^**-1**^**)**	0.0739 (0.0635, 0.0826)	0.1256 (0.1046, 0.1361)	0.0643 (0.0550, 0.0716)	0.5839 (0.5297, 0.6303)
***k***_***g***_ **(h**^**-1**^**)**	1.0873 (0.9427, 1.4609)	1.0873 (0.7064, 1.2345)	1.6816 (1.5103, 1.9171)	0.804 (0.5940, 1.0578)
***B*(h**^**-1**^**)**	1.8196 (1.6446, 1.9935)	1.8196 (1.6446, 6.2878)	3.1388 (2.9033, 3.2406)	3.1388 (2.9033,9.5225)
***C***	3.53E-06 (8.21E-7,1.61E-5)	3.53E-06 (8.21E-7, 0.0125)	1.93E-9 (1.00E-9, 1.18E-08)	1.93E-9 (1.00E-9, 1.18E-08)
***K***_***I***_	0	0.1189 (0.8609, 4.8592)	0	0.1060 (0.1060, 2.3517)
***μ***_***V***_**(h**^**-1**^**)**	0.2322 (0.1753, 0.2989)	0.3373 (0.2904, 0.6067)	6.19E-7 (1.00E-8, 0.0163)	0.2000 (0.1577, 0.2536)

*Epithelial cells alone at ALI

**1:40 ratio of immune:epithelial cells at ALI

We observed that the model did not fit the co-culture data well when we used the values estimated for the total number of spores that failed to germinate (*S*_*s*_) and death rates of vegetative bacteria (*μ*_*V*_) derived from the epithelial cell-only dataset. Based on the data, this was expected for *S*_*s*_ because the number of spores that fail to germinate is higher in the co-culture systems than in the epithelial cell-only systems: 1.7 and 9.1 times higher in the rabbit and human co-culture systems, respectively.

After fitting the data, we calculated the lag time for proliferation using the formula, ln(1+1/*C*)/(*B*/(1+*K*_*I*_)). In the rabbit system, the proliferation lag times were 6.9 and 7.72 hours in the absence and presence of immune cell inactivation, respectively. The corresponding lag times in the human system were 6.39 and 7.07 hours, respectively. Thus, our *in vitro* PBBK model is able to correctly capture the longer delay in bacterial proliferation through the inactivation parameter, *K*_*I*_. The effect of *K*_*I*_ on the model results is provided as supporting information in S1 Fig in the supplemental information.

### *In Vivo* PBBK model calibration for rabbits

The *in vivo* PBBK model incorporates the germination and proliferation growth kinetics from the *in vitro* PBBK model, and the kinetics for mucociliary clearance and airway-body spore/bacteria transfer from the PBBK model by Gutting [4]. We calibrated the *in vivo* model to the total bacteria (*S*_*a*_ + *V*_*a*_ + *S*_*b*_ + *V*_*b*_) growth data from rabbit *in vivo* studies (see Table 8 of reference, Gutting 2014), where the data was collected up to 36 hours from rabbits exposed to an initial inhaled dose of 66.4 x LD_50_ *B*. *anthracis* Ames spores estimated from Gutting 2014 [4]. The total deposited dose in Gutting’s experiments (Gutting 2014) was 6.2 x 10^5^ spores, which was 9.2% of the total inhaled spore dose. Thus, based on an LD_50_ value of 101,500 spores [13], we estimate the total inhaled spore dose was 66.4 x LD_50_. We fitted the model to the data by fixing the spore germination rate (***k***_***g***_ ***= K***_***g***_), and maximal growth rate (***B***) from the *in vitro* parameter estimates from our experiments with the non-lethal Sterne strain and re-calibrating the physiological adjustment state (***C***) and inactivation capacity of the immune cells (***K***_***I***_) for the lethal Ames strain.

**Fig 7:**
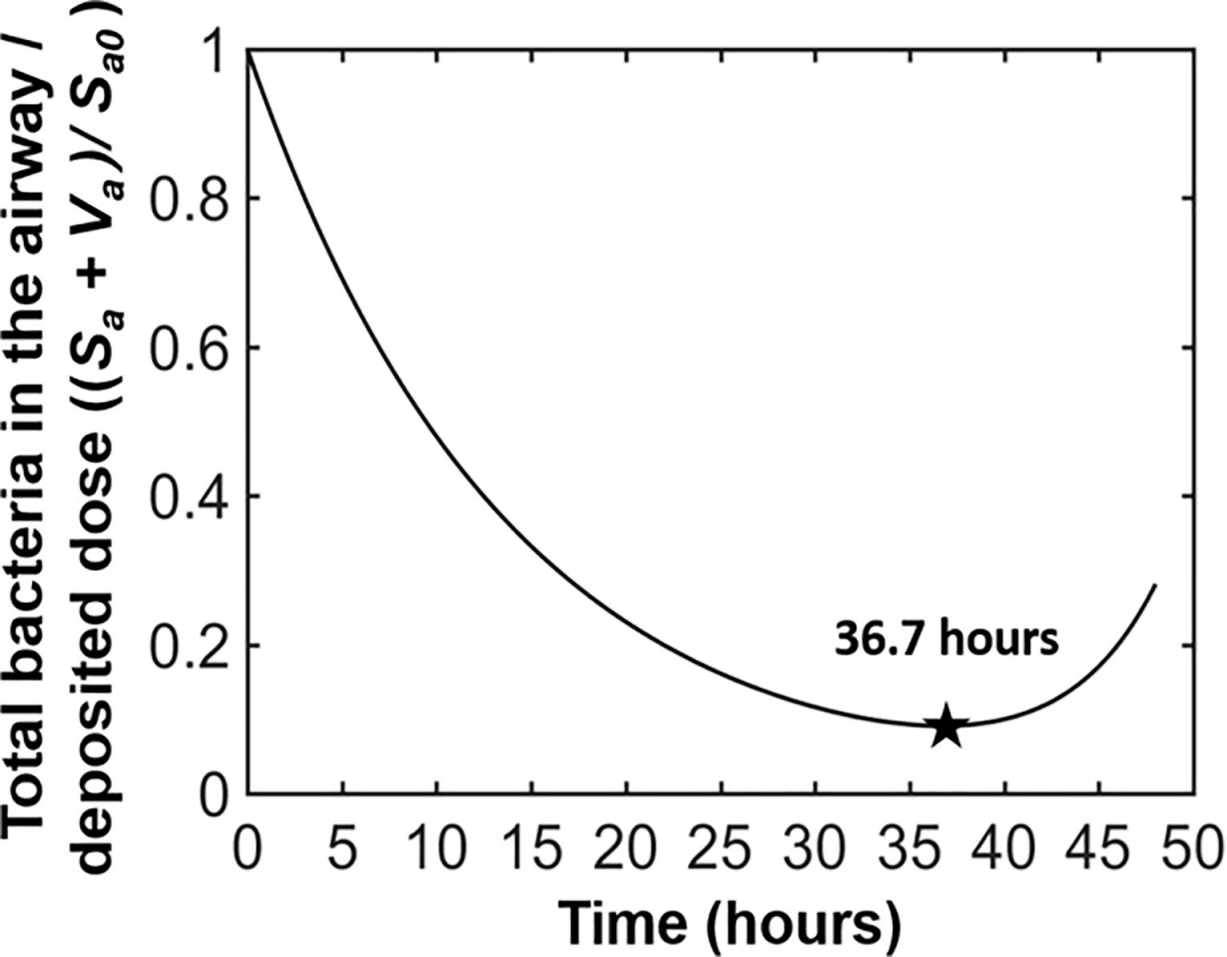
The length of time for the bacteria in the body to return to the airway and then proliferate following an inhalation event based on the *in vivo* PBBK model for NZW rabbits.

The final values of the *in vivo* model parameters for rabbits are provided in Table 5. The results of the model fitting for rabbits are provided in S2 Fig, and the predictions for each compartment of the *in vivo* PBBK model are provided in S3 Fig. Our results show that over time, spores germinate in the body compartment, giving rise to vegetative cells followed by proliferation of vegetative cells. Running the model for longer than 36 hours reveals an increase in the number of total bacteria in the airways (Fig 7). As shown in Fig 7, the time at which the bacteria in the airways begin to increase is 36.7 hours. Gutting’s (2014) model [4] predicted this time as 45.1 hours, which was referred to as the mean time to death (MTD) because it was very close to the MTD value (46.9 hours) observed for rabbits exposed to lethal inhaled doses of Ames spores previously reported by Yee *et al*. [14].

**Table 5:** *In vivo* PBBK model parameters for NZW rabbits.

Parameter	Value (rabbit)
***K***_***mc***_ (h^-1^)	0.0628^a^
***K***_***trans***_ (h^-1^)	0.0107^a^
***K***_***sec***_ (h^-1^)	0.0001^a^
***K***_***g***_ (h^-1^)	1.0873^b^
***B*** (h^-1^)	1.8196^b^
***C***	6.78^c^
***K***_***I***_	6.19^c^
***K***_***k***_ (h^-1^)	0.0431^a^

a) Gutting 2014 [4]

b) Estimated by calibrating our *in vitro* PBBK model with data for *B*. *anthracis* Sterne in NZW rabbit co-culture system at ALI

c) Estimated by calibrating our *in vivo* PBBK model with bacterial growth data for *B*. *anthracis* (Ames strain) in NZW rabbits from Gutting (2014) [4]

### *In vivo* model predictions for human

Based on how the re-calibrated parameter values (for *C* and *K*_*I*_) linearly scaled with respect to the *in vitro* values for the rabbits, the corresponding parameters for the human were proportionally scaled to make an *in vivo* PBBK model prediction for the human. We applied the same transport parameter values (*K*_*mc*_, *K*_*trans*_, *K*_*sec*_, and *K*_*k*_) from the NZW rabbit as the best estimate to the human system since it is not known how the these parameters would scale between a rabbit and a human. The final values of the estimated model parameters for humans are provided in Table 6.

**Table 6:** *In vivo* PBBK model parameters for human.

Parameter	Value
***K***_***mc***_ (h^-1^)	0.0628^a^
***K***_***trans***_ (h^-1^)	0.0107^a^
***K***_***sec***_ (h^-1^)	0.0001^a^
***K***_***g***_ (h^-1^)	0.804^b^
***B*** (h^-1^)	3.1388^b^
***C***	0.00371^c^
***K***_***I***_	5.5184^c^
***K***_***k***_ (h^-1^)	0.0431^a^

a) Gutting 2014 [4]

b) Estimated by calibrating the *in vitro* PBBK model with data for *B*. *anthracis* Sterne in human co-culture system at ALI

c) Scaled up from *in vitro* values based on the *in vitro* to *in vivo* scaling observed for rabbits.

**Fig 8:**
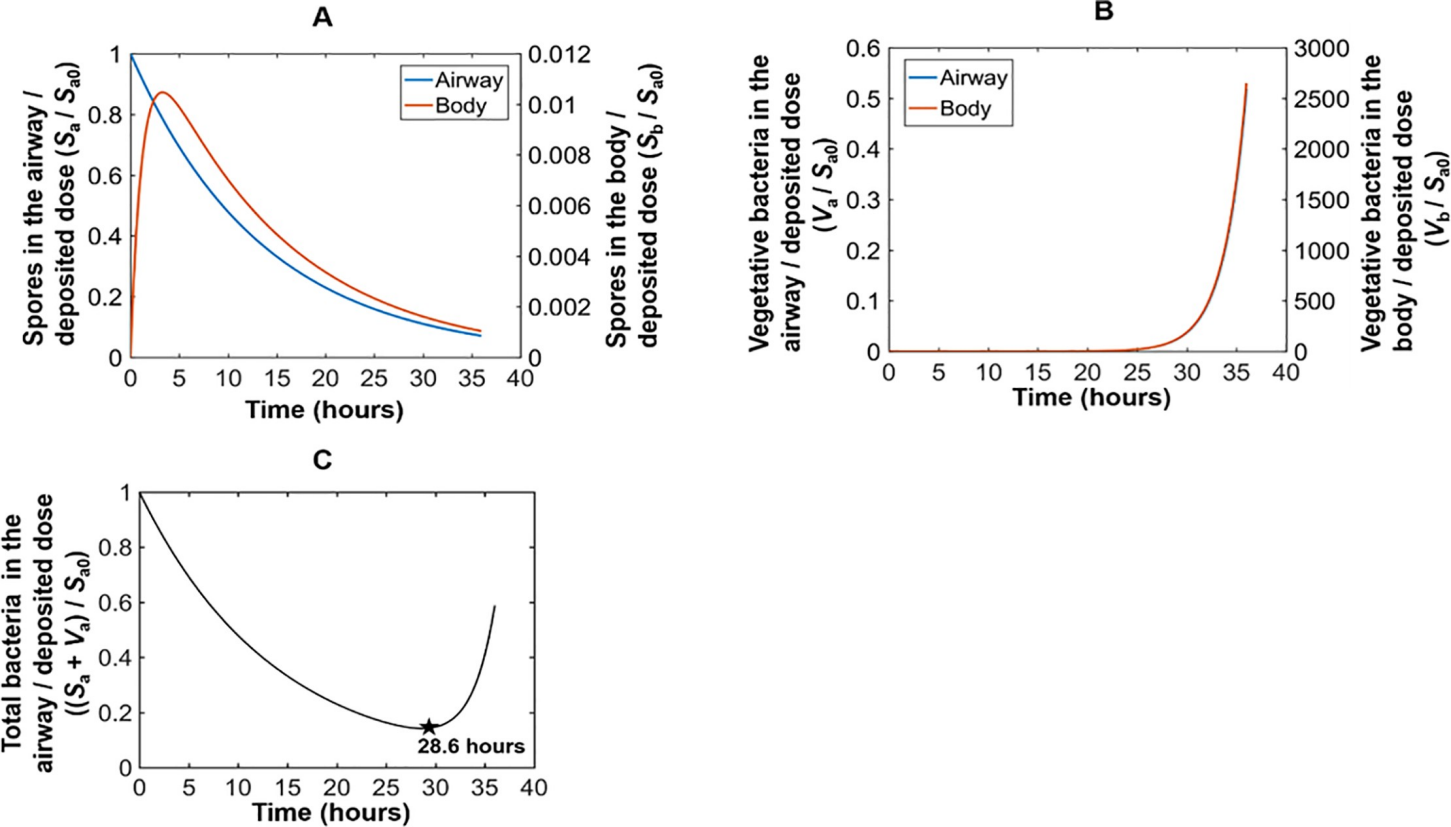
Predictions of the *in vivo* PBBK model for human. Spores (A) and vegetative bacteria (B) over time in each compartment of the *in vivo* PBBK model. (C) Total bacteria over time in the airway. The blue line for the vegetative bacteria in the airway (B) exists but is not visible on the plot because it is almost overlapping with the red line of the vegetative bacteria in the body.

The parameter values (in Table 5) were applied in the *in vivo* PBBK model to predict the total spore fraction and fraction of vegetative bacteria versus time for the body and airway compartments in the human. The results are shown in Fig 8A. The model predicted that the time at which the bacteria in the airways begin to increase following exposure without preventative measures is 28.6 hours (Fig 8B), which is earlier than the 36.7 hours predicted for the NZW rabbit. In addition, the ratio of total bacteria to the initial spore dose at 36 hours post exposure is 57.4 in the NZW rabbit compared to 2,650 in the human. These simulation results, derived from our *in vitro* experimental data in rabbits and humans and previously published *in vivo* data in rabbits indicate that disease progression is likely to be faster in humans than in NZW rabbits under comparable total deposited dose conditions.

### Relationship between mean time to death and deposited dose

Based on dose-response and reported MTD data from rabbit studies by the EPA [15, 16], we found that MTD and inhaled dose are correlated according to the following power-law equation, $MTD=24.765D_{0}^{-0.124}$ (S4 Fig), where D_o_ is the inhaled dose. Additional data points will be useful to confirm this power-law behavior. Based on the power-law equation, the MTD for the inhaled dose of 66.4 LD_50_ is 84.59 hours (3.52 days), which is 1.8 times higher than the value (46.9 hours) reported by Yee *et al*. [14] and used by Gutting [4] to associate the critical time point to MTD. The doses in the EPA dose-response data, from which we derived the power-law relationship, range from 0.25 LD_50_ to 81.53 LD_50_; the corresponding % lethal response range from 40% to 100% (see S5 Fig).

We note that the MTD value of 46.9 hours reported by Yee et al. is an average of the MTD values observed for the lethal doses that ranged from 51.1 to 313.8 LD_50_) [14]. No correlation is observed between MTD and dose in this dose range, which is within the range that showed 100% lethality in the rabbit dose-response data compiled by Gutting *et al*. [17]. Hence, the lack of correlation between MTD and dose in the 100% lethal dose range from Yee et al.’s data and the power-law correlation in the 40–100% lethal dose range based on EPA data, suggests that there is uncertainty with estimating an MTD value for the 100% lethal dose range. Moreover, it should be noted that the reported MTD value at each tested dose by Yee et al. was based on a single rabbit; it was not based on replicate data as in the EPA dataset. Variability in MTD values should be expected for a single dose group with multiple rabbits, which is not captured in Yee et al.’s data. Given the between-dose variability of MTD in the 100% lethal dose range, adding in the expected within-dose MTD variability may or may not remove the uncertainty associated with identifying a MTD value in the 100% lethal dose range.

## Discussion

Our primary goal was to establish a computational PBBK model describing the key events leading to virulence of inhaled *B*. *anthracis* spores between humans and experimental animals. We parameterized the PBBK model using data obtained from the Sterne strain, which is expected to be a reasonable surrogate for more virulent strains in terms of spore germination and proliferation, but may overestimate the immune cell inactivation parameter. The PBBK model provides the foundation for improved prediction of virulence, time to death, and dose-response relationships for inhaled *B*. *anthracis* spores in both animals and humans. With the computational model established, future studies can now begin refining the model parameters using data obtained from virulent strains defined under identical experimental conditions.

### Extrapolation of Dose-response curves for human

The current approach for extrapolation of rabbit-to-human dose-response curve involves transforming rabbit inhaled doses into human-equivalent doses using existing computational 3D lung dosimetry models and species-to-species dose scaling relationships [6, 7]. This approach is based upon the assumption that humans will respond the same way as rabbits at the human-equivalent doses. One such extrapolation is shown in Supporting Information (S6 Fig) using the inhaled concentration-response datasets for rabbits collated by Gutting et al (2016). The data show little/no difference in lethality following a single acute exposure, or two weeks of repeated exposures in rabbits when the data are normalized to a “total inhaled dose”. Therefore, for any atmospheric concentration and single exposure duration (< 2 weeks), the total inhaled dose can be calculated based on species ventilation, and then multiplied by the deposited dose fraction to determine the total deposited dose profile (see Supporting Information, S2 and S3 Tables). The deposited dose fraction will be different for rabbit and humans because of species differences in lung structure and breathing profiles. Using Multi-Path Particle Dosimetry (MPPD v. 3.04), we estimated the total deposited dose fraction is higher in humans than in rabbits: 0.135 for the human and 0.054 for the rabbit (see S5 Fig). MPPD takes into account the size and density of the particles, the structure of the lung, and the exposure scenario (values are provided in S1 Table). Based on MPPD-predicted dose fraction and published scaling relationships (Asgharian et al. 2016) [6], humans are more sensitive than NZW rabbits if the dose-response relationship is assumed to be the same for both rabbit and human (S6 Fig).

Although species differences for the deposited dose fraction of inhaled spores can be estimated and taken into account, accurate and reliable rabbit-to-human dose-response extrapolations (and an infectious dose estimation) for a human require knowledge about the growth and virulence characteristics of the pathogen in both rabbits and humans. Therefore, the overarching goal for the current work is to establish an approach that combines a physiologically-relevant *in vitro* lung ALI co-culture system with *in silico* PBBK models to predict a dose-response curve (and in turn the infectious dose) for humans exposed to any respiratory pathogen, such as *B*. *anthracis*. In this work, the rabbit-to-human extrapolation is based upon the assumption that the *in vitro*-*in vivo* scale up of the parameters [the physiological adjustment state (***C***) and inactivation capacity of the immune cells (***K***_***I***_), which are likely to be affected by the pathogenicity of Ames vs. Sterne strains were used to fit the rabbit *in vivo* data] applies to humans. The other parameters come directly from the *in vitro* experiments conducted with human cells (just like the rabbits). This approach, however, has to be fully validated after incorporating dose-dependent growth effects in the *in vitro* and *in vivo* PBBK models.

The current *in vivo* PBBK model is linear in S (*S*_*a*_ and *S*_*b*_) and V (*V*_*a*_ and *V*_*b*_), and the data used for estimating the parameters, physiological adjustment state (*C*) and inactivation capacity of the immune cells (*K*_*I*_), are only available in the 100% lethal dose range from *in vivo* studies with NZW rabbits. Therefore, the fitted model can only be used to predict the growth of bacteria under 100% lethal dose conditions; it cannot be used for predicting dose-dependent growth under non-lethal conditions which will require additional studies. If the correlation between the time at which the total bacteria increases in the airway compartment of the *in vivo* PBBK model (critical time point) and experimental MTD exists also in the sub-lethal dose range, then a model incorporating dose-dependent kinetics can be used to predict the critical time points and in turn estimate an MTD for each dose.

## Conclusion

The *in vitro* lung ALI system and the *in silico* PBBK models, developed in this work for *B*. *anthracis*, provide a framework for predicting dose-response curves (and subsequently an infectious dose) for humans exposed to respiratory pathogens. The lung ALI system and the *in vitro* PBBK model capture the physiologically relevant growth processes for *B*. *anthracis* (germination, proliferation, immune inactivation). The reproducibility of the data collected from the ALI system can be tested by ensuring that the parameter values of the *in vitro* PBBK model are reproducible within experimental variability in the measured number of spores and vegetative bacteria. Thus, the current ALI system and the *in vitro* PBBK model can readily be applied to characterize the growth and virulence of respiratory pathogens in rabbits and humans. With the accuracy and reproducibility of the *in vitro* measurements that inform the computational framework now established, studies focused on capturing the same model parameters for the Ames strain can be investigated to improve species comparisons and prediction of virulence in humans.

## Supporting information

S1 Fig **Effect of immune cell inactivation parameter, *K*_*I*_, on the proliferation of vegetative bacteria in rabbit (A) and human (B) co-culture systems.** Time-course data of vegetative bacteria counts (*V*), normalized by the actual spore dose, *S*_0_. Red solid circles represent experimental data points and lines represent model output for each *K*_*I*_ value.
(TIF)

S2 Fig Total bacteria in the rabbit body versus time using the calibrated *in vivo* PBBK model. S_a0_ refers to the deposited dose at time zero (6.2 x 10^5^ spores). The red circles are the experimental values from Gutting [1] and the blue curve is the fit to the data.
(TIF)

S3 Fig Estimates of spore fraction and vegetative bacteria fraction over time in each compartment of the *in vivo* PBBK model for the rabbit. (TIF)

S4 Fig Correlation between mean time to death and inhaled dose of *B. anthracis* Ames spores for rabbits, based on data reported by EPA. (TIF)

S5 Fig Deposited dose fraction of *B. anthracis* spores in the head, tracheal-bronchial (TB) and pulmonary (P) regions in rabbits and humans. (TIF)

S6 Fig Percent mortality versus number of inhaled *B. anthracis* Ames spores for rabbit and human (extrapolated). (TIF)

S1 Table Input data used for the MPPD (v. 3.04) calculations. (DOCX)

S2 Table Deposited spore dose in a rabbit after an hour of exposure to various atmospheric concentrations. (DOCX)

S3 Table Deposited spore dose in a human after an hour of exposure to various atmospheric concentrations. (DOCX)
