# Supplementary figures and images for: An integrated experimental-computational approach for predicting virulence in New Zealand white rabbits and humans following inhalation exposure to Bacillus anthracis spores
Source: PLoS One. 2019 Jul 1;14(7):e0219160. doi: 10.1371/journal.pone.0219160 (PMC6602573; doi:10.1371/journal.pone.0219160)

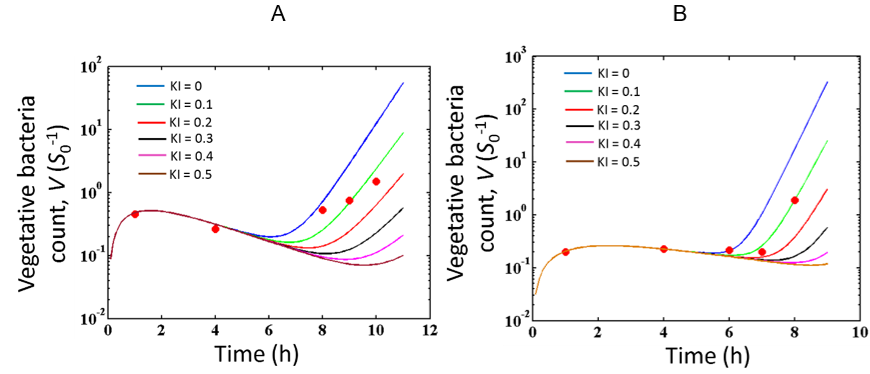

Supplement: S1 Fig — Effect of immune cell inactivation parameter, KI, on the proliferation of vegetative bacteria in rabbit (A) and human (B) co-culture systems. Time-course data of vegetative bacteria counts (V), normalized by the actual spore dose, S0. Red solid circles represent experimental data points and lines represent model output for each KI value. (TIF) [file pone.0219160.s001.tif]

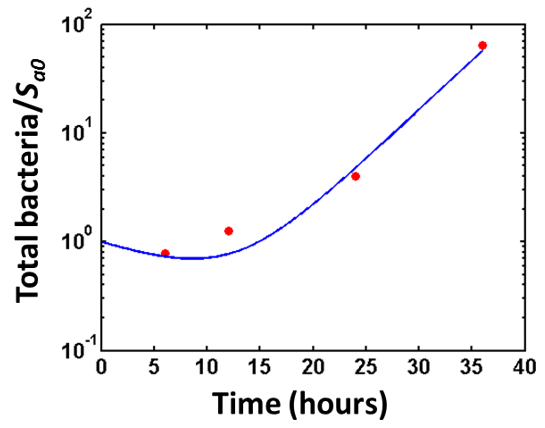

Supplement: S2 Fig — Sa0 refers to the deposited dose at time zero (6.2 x 10^5^ spores). The red circles are the experimental values from Gutting [1] and the blue curve is the fit to the data. (TIF) [file pone.0219160.s002.tif]

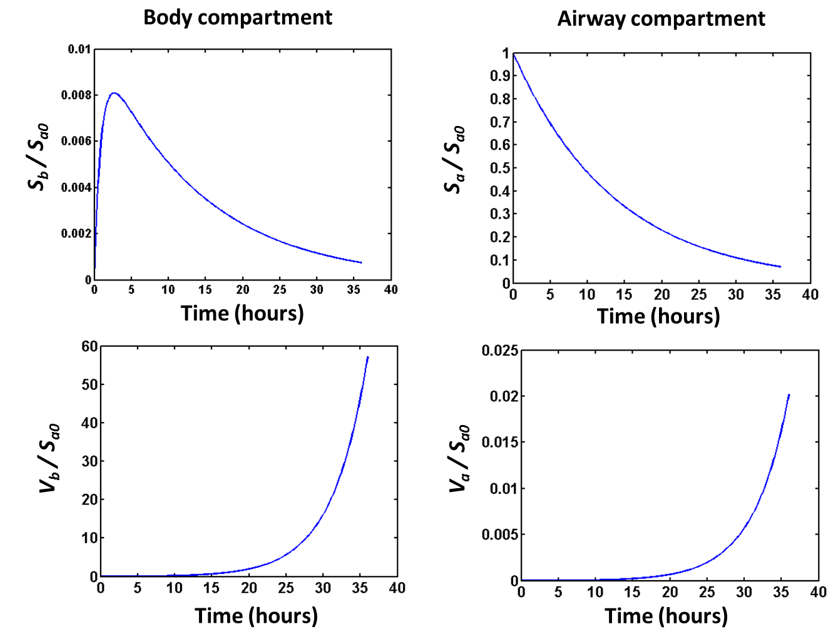

Supplement: S3 Fig — (TIF) [file pone.0219160.s003.tif]

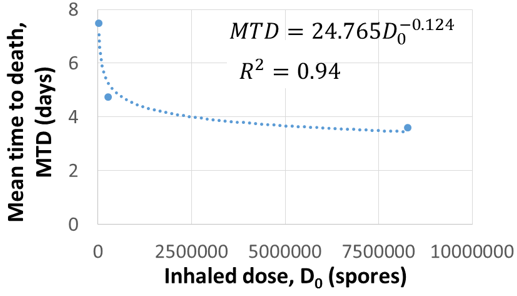

Supplement: S4 Fig — (TIF) [file pone.0219160.s004.tif]

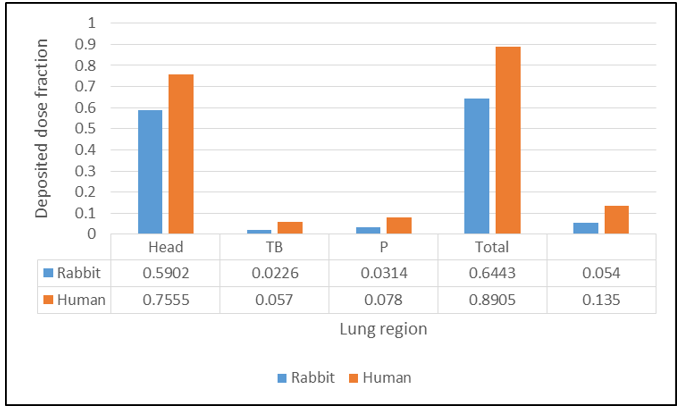

Supplement: S5 Fig — (TIF) [file pone.0219160.s005.tif]

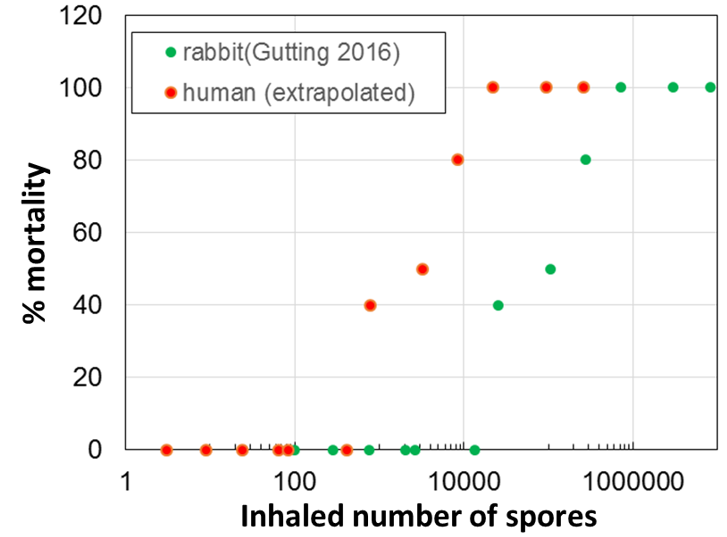

Supplement: S6 Fig — (TIF) [file pone.0219160.s006.tif]
