# Supplementary material for: An integrated experimental-computational approach for predicting virulence in New Zealand white rabbits and humans following inhalation exposure to Bacillus anthracis spores
Source: PLoS One. 2019 Jul 1;14(7):e0219160. doi: 10.1371/journal.pone.0219160 (PMC6602573; doi:10.1371/journal.pone.0219160)
Supplement: S1 Table — (DOCX) [file pone.0219160.s007.docx]

| Model/parameter | Rabbit | Human |
| --- | --- | --- |
| *Lung parameters* | | |
| Lung geometry | Asymmetric | PNNL Asymmetric |
| Functional residual capacity volume (mL) | 37.80 | 3300 |
| Upper respiratory tract volume (mL) | 7.02 | 50 |
| *Exposure scenario* | | |
| Body orientation | Upright | Upright |
| Breathing route | Nasal | Nasal |
| Tidal volume (mL) | 29.11 | 625.00 |
| Breathing frequency (min^-1^)^a^ | 80 | 20 |
| Inspiratory Fraction^b^ | 0.99 | 0.99 |
| Pause Fraction | 0.00 | 0.00 |
| *Particle Properties* | | |
| Diameter (µm) | 1.0 | 1.0 |
| GSD | 1.0 | 1.0 |
| Concentration (mg/m^3^) | 1.0 | 1.0 |
| Density (g/cm^3^) | 1.0 | 1.0 |
| Aspect Ratio | 1.0 | 1.0 |
|  |  |  |
