# Supplementary material for: An integrated experimental-computational approach for predicting virulence in New Zealand white rabbits and humans following inhalation exposure to Bacillus anthracis spores
Source: PLoS One. 2019 Jul 1;14(7):e0219160. doi: 10.1371/journal.pone.0219160 (PMC6602573; doi:10.1371/journal.pone.0219160)
Supplement: S2 Table — (DOCX) [file pone.0219160.s008.docx]

| **atmospheric particle conc (mg/m^3^)** | **inhaled particle mass (mg)** | **inhaled number of spores** | **deposited mass (µg)** | | | **deposited number of spores** |
| --- | --- | --- | --- | --- | --- | --- |
|  |  |  | **TB** | **P** | **TB + P** |  |
| 2.20E-08 | 3.07402E-09 | 5.87E+00 | 6.96E-08 | 9.65E-08 | 1.66E-07 | 3.17E-01 |
| 2.20E-07 | 3.07402E-08 | 5.87E+01 | 6.96E-07 | 9.65E-07 | 1.66E-06 | 3.17E+00 |
| 2.20E-06 | 3.07402E-07 | 5.87E+02 | 6.96E-06 | 9.65E-06 | 1.66E-05 | 3.17E+01 |
| 2.20E-05 | 3.07402E-06 | 5.87E+03 | 6.96E-05 | 9.65E-05 | 1.66E-04 | 3.17E+02 |
| 2.20E-04 | 3.07402E-05 | 5.87E+04 | 6.96E-04 | 9.65E-04 | 1.66E-03 | 3.17E+03 |
| 2.20E-03 | 0.000307402 | 5.87E+05 | 6.96E-03 | 9.65E-03 | 1.66E-02 | 3.17E+04 |
| 2.20E-02 | 0.003074016 | 5.87E+06 | 6.96E-02 | 9.65E-02 | 1.66E-01 | 3.17E+05 |
| 2.20E-01 | 0.03074016 | 5.87E+07 | 6.96E-01 | 9.65E-01 | 1.66E+00 | 3.17E+06 |
