# Supplementary material for: An integrated experimental-computational approach for predicting virulence in New Zealand white rabbits and humans following inhalation exposure to Bacillus anthracis spores
Source: PLoS One. 2019 Jul 1;14(7):e0219160. doi: 10.1371/journal.pone.0219160 (PMC6602573; doi:10.1371/journal.pone.0219160)
Supplement: S3 Table — (DOCX) [file pone.0219160.s009.docx]

| **atmospheric particle conc (mg/m^3^)** | **inhaled particle mass (mg)** | **inhaled number of spores** | **deposited mass (µg)** | | | **deposited number of spores** |
| --- | --- | --- | --- | --- | --- | --- |
|  |  |  | **TB** | **P** | **TB + P** |  |
| 2.2E-09 | 1.65E-09 | 3.15E+00 | 9.41E-08 | 1.29E-07 | 2.23E-07 | 4.26E-01 |
| 2.20E-08 | 1.65E-08 | 3.15E+01 | 9.41E-07 | 1.29E-06 | 2.23E-06 | 4.26E+00 |
| 2.20E-07 | 1.65E-07 | 3.15E+02 | 9.41E-06 | 1.29E-05 | 2.23E-05 | 4.26E+01 |
| 2.20E-06 | 1.65E-06 | 3.15E+03 | 9.41E-05 | 1.29E-04 | 2.23E-04 | 4.26E+02 |
| 2.20E-05 | 1.65E-05 | 3.15E+04 | 9.41E-04 | 1.29E-03 | 2.23E-03 | 4.26E+03 |
| 2.20E-04 | 1.65E-04 | 3.15E+05 | 9.41E-03 | 1.29E-02 | 2.23E-02 | 4.26E+04 |
| 2.20E-03 | 1.65E-03 | 3.15E+06 | 9.41E-02 | 1.29E-01 | 2.23E-01 | 4.26E+05 |
| 2.20E-02 | 1.65E-02 | 3.15E+07 | 9.41E-01 | 1.29E+00 | 2.23E+00 | 4.26E+06 |
| 2.20E-01 | 1.65E-01 | 3.15E+08 | 9.41E+00 | 1.29E+01 | 2.23E+01 | 4.26E+07 |
